# Clear Cell Sarcoma of Soft Tissues: Radiological Analysis of 14 Patients—MRI Findings Related to Metastatic Disease

**DOI:** 10.3390/diagnostics15081027

**Published:** 2025-04-17

**Authors:** Paola Di Masi, Marco Colangeli, Mario Simonetti, Giuseppe Bianchi, Alberto Righi, Gabriele Bilancia, Emanuela Palmerini, Amandine Crombé, Paolo Spinnato

**Affiliations:** 1 Diagnostic and Interventional Radiology, IRCCS Istituto Ortopedico Rizzoli, 40136 Bologna, Italy; 2 Department of Orthopaedic Oncology, IRCCS Istituto Ortopedico Rizzoli, 40136 Bologna, Italy; 3 Department of Pathology, IRCCS Istituto Ortopedico Rizzoli, 40316 Bologna, Italy; 4 Osteoncology, Soft Tissue and Bone Sarcomas, Innovative Therapy Unit, IRCCS Istituto Ortopedico Rizzoli, 40136 Bologna, Italy; 5 Sylvester Comprehensive Cancer Center, University of Miami, Miami, FL 33124, USA; 6 Miller School of Medicine, University of Miami, Miami, FL 33124, USA; 7 Department of Musculoskeletal Imaging, Pellegrin University Hospital, 33076 Bordeaux, France

**Keywords:** magnetic resonance imaging, soft tissue sarcomas, diagnosis, prognosis, sarcoma, clear cell sarcoma

## Abstract

**Background/Objectives:** Clear cell sarcoma (CCS) is a very uncommon, aggressive soft-tissue sarcoma (STS) with a dismal prognosis. In the current literature, there are very limited data focused on the radiological features of CCS. Our study’s objective was to describe CCS pre-treatment’s peculiar imaging characteristics (MRI above all) and to assess if some radiologic features may predict patients’ outcomes with regard to the occurrence of distant metastases. **Methods**: Our single-center experience includes all the patients with a histological diagnosis of CCS and available radiological and clinical data: 14 patients (8M, 6F, mean age 39.4 years old) were included. The available pre-treatment MRI or contrast-enhanced computed tomography (CECT) studies were examined using an analytical grid that incorporated characteristics from the most recent STS research. The occurrence of metastatic disease was matched with radiological features from baseline imaging studies. **Results**: MRI was available in 13 patients and CECT in 1 patient. The mean longest diameter (LD) was 50.5 mm ± 29.2. In 10 cases (71.4%), the tumor was deeply seated. MRI revealed a slightly high signal intensity (SI) on T1-WI and a high SI on T2-WI in every subject. At baseline, metastases were already present in 5/14 (35.7%) patients, 3 more developed metastases during the following 5 years (8/14, 57.1%), and 2 additional developed late-onset metastasis after more than 5 years from the diagnosis (total of 10/14 metastatic patients 71.4%). LD and metastasis at diagnosis were significantly correlated (Pearson correlation = 72%, *p*-value = 0.004). A pre-treatment LD > 4 cm was significantly associated with the development of distant metastases within 5 years from diagnosis and in subsequent follow-up (*p* = 0.0003). LD > 4 cm represents an increase in risk of metastatic disease within 5 years and during the course of follow-up (OR = 195.00, 95%CI: 3.36–11285.55, *p* = 0.01). The presence of MRI signs of macroscopic necrosis represented an increase in risk of metastatic disease within 5 years (OR = 15.00, 95%CI: 1.03–218.31—*p* = 0.04). **Conclusions:** The identification of MRI features of aggressive biology is a key element for an early referral to sarcoma centers. In our study, a LD > 4 cm and the presence of MRI signs of macroscopic necrosis at the baseline images resulted in being a predictor of metastatic disease.

## 1. Introduction

### 1.1. General Characteristics, History and Epidemiology

Clear cell sarcoma (CCS) is an extremely rare malignant tumor that accounts for less than 1% of soft tissue tumors and often involves distal tendons and aponeuroses [1,2,3].

Its histogenesis has been the subject of various investigations since Enzinger initially described it as a unique clinico-pathological entity in 1965 [4]. Because of its close histological relationship to malignant melanoma (presence of melanin, ultrastructural evidence of melanosomes, and immunohistochemical staining for the S-100 protein and the melanoma-associated antigen HMB-45), Chung and Enzinger proposed the name malignant melanoma of the soft tissue [2,4,5,6].

More modern molecular genetic characterization of the two diseases has led to the conclusion that CCS and malignant melanoma constitute two distinct entities. The t [12;22] [q13;q12] translocation resulting in the production of the chimeric EWSR1/ATF1 and EWSR1/CREB1 genes is only found in CCS and not in cutaneous malignant melanoma; conversely, activating mutations in the kinase domain of the BRAF gene are only found in cutaneous malignant melanoma and not in CCS [2,7]. Similar to many other soft tissue sarcomas, it has a deep soft tissue initial site that does not invade the skin. CCS usually metastasizes to the lymph nodes and lungs [8,9].

Compared to the extensive literature on the histopathological nature of the disease, knowledge of the clinical and radiological features of CCS is still limited to a few case reports and case series. The main obstacle to understanding the clinical behavior of CCS is its rarity. Even among the studies conducted at major centers, most have involved fewer than a few dozen patients. To date, the largest multiple-center radiological study consisted of 21 patients with MRI but included a restricted analysis grid and no assessment of correlations between imaging and patient outcome [10]. These limitations have hindered the definition of prognostic factors and well-founded treatment protocols for CCS [2].

CCS is typically a relatively small (<5 cm), deep lesion, often juxtaposed to tendons, fascia, or aponeurosis. It is more common in Caucasians than in African Americans or Asians, with no gender predilection [1,11,12]. The age of patients varies with most cases in the literature reported between 20 and 40 years of age and a limited number of cases beyond the fourth decade [13,14]; 2% have occurred in children younger than 10 years of age [14]. The most common localization sites are the foot and ankle and, secondarily, the knee; other sites in the skeleton are the upper extremities (wrist, hand) [3,14]. Localization to the penis, retroperitoneum, kidney (especially in children), pancreas, and gastrointestinal tract has been reported [3,14]. Clear cell sarcoma of the bone may be primary or metastatic. The distinction between clear cell sarcoma and melanoma, especially in the case of metastatic disease at diagnosis with an unknown primary tumor, often creates a diagnostic challenge [15].

### 1.2. Anatomical Pathology and Genetic and Molecular Patterns

**Figure 1:**
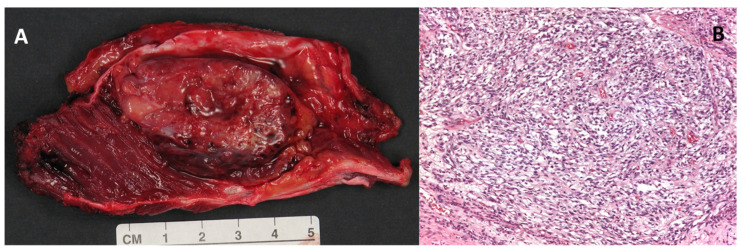
(**A**) Macroscopic appearance of a large (5 cm) clear cell sarcoma of the soft tissues after surgical excision. (**B**) Histologic example of clear cell sarcoma, composed of tumor cells arranged in a short fascicular or a solid sheet-like growth pattern delineated by fibrous septa (magnification 100×).

Macroscopically, the clear cell sarcoma is a grey-brown, firm, circumscribed mass (Figure 1, Panel A). Microscopically, the tumor is highly infiltrating and organized in small nests and compact fascicles of neoplastic cells along the dense fibrous connective tissue of the tendons, fascia, and aponeurosis (Figure 1, Panel B) [1,15,16].

The cell nests are divided into lobules by a fine framework of collagenous fibers of varying thickness that is contiguous to the fibrous connective tissue of the adjacent tendons and aponeurosis. The cells show minimal or absent pleomorphism. Mitotic figures are scarce in accordance with the slow-growing behavior of the tumor. Scattered multinucleated giant cells are often present, and areas of necrosis and melanin pigment can be identified [4,17]. The neoplastic cells have a polygonal to fusiform morphology, with clear or pale, eosinophilic to amphiphilic cytoplasm, and have uniform, round or ovoid and vesicular nuclei in a central position with prominent basophilic nucleoli, similar to those of melanoma. The clear cell appearance is due to the accumulation of glycogen [1,4].

CCS is considered a neoplasm derived from neural crest cells [3,16]. Despite its kinship with malignant melanoma (melanotic differentiation, ultrastructural evidence of melanosomes, and high frequency of metastases), CCS is genetically different from malignant melanoma or other neuroectodermal or mesenchymal-derived tumors due to the translocation of EWSR1, an uncommon genetic event in melanoma [2,7].

Immunohistochemically, clear cell sarcoma shows a phenotype identical to that of conventional melanoma, characterized by strong expression of S100 protein in 100% of cases and expression of HMB-45, Melan-A, and MiTF in 81–97% of cases. It may also express neuroendocrine and/or nerve sheath neoplasia markers, including synaptophysin, CD56, and CD57 in 21–75% of cases; CD34 is generally negative [1,15].

The reciprocal translocation t (12;22) (q13;q12) is observed in more than 90% of clear cell sarcoma cases by chromosome analysis, reverse transcriptase polymerase chain reaction (RT-PCR) and in situ hybridization (FISH). This translocation results from the fusion of a portion of the activating transcription factor gene (ATF-1) on the long arm of chromosome 12 (12q13) and the Ewing’s sarcoma oncogene R1 (EWSR1) on chromosome 22 (22q12). It has been shown that the EWSR1/ATF1 fusion protein is able to bind to and activate the melanocyte-specific microphthalmia-associated transcription factor (MiTF); this, in the presence of the sox10 transcription factor, determines the expression of the melanocytic phenotype and regulates the growth and survival of clear cell sarcoma. In addition to the diagnostic translocation t (12;22) (q13;q12), polysomy of chromosome 8 has been observed in many cases of clear cell sarcoma as a secondary abnormality [18,19].

The FNLCC grading system, the standard system for histological grading in all STS, is not applicable to CCS. Indeed, currently, CCS has no validated histological grading system to be applied.

### 1.3. Imaging and Clinical Features

The diagnosis of clear cell sarcoma is difficult due to its rarity and lack of specific clinical and radiological features. Clinical manifestations vary from the absence of symptoms to pain/tenderness. Usually, CCS does not show any symptoms until it progresses.

An accurate diagnosis depends on the physician’s level of experience at the time of presentation, and timely referral to a specialized institution is essential. The tumor is slow-growing, and often, several years elapse between its appearance and diagnosis. The patient frequently undergoes inadequate surgical excision with only later detection of its malignancy [20]. Some studies have correlated a diameter greater than 5 cm with an increased metastatic risk [21,22]. Approximately 30% of patients have a tumor that is already locally advanced or metastatic at diagnosis, and even in the case of radical surgery, a large proportion of patients will suffer recurrence, reflecting the aggressive nature of the tumor [1]. CCS is typically resistant to anthracycline chemotherapy and radiotherapy. Prognosis is often poor, with a median 5-year survival of 67%, but this drops to 20% in the case of metastatic disease [23].

Imaging plays a very important role in the evaluation of soft tissue tumors in order to analyze the specific features of the tumor and differentiate CCS from other pathologies. Radiography and even more CT scans can be useful in the evaluation of possible, although very rare, calcifications and bone erosions. Ultrasonography and MRI allow a more accurate study of the tissue component of the lesion and the involvement of surrounding soft tissues.

If the patient has a history of trauma, the mass may be confused with a hematoma. Clear cell sarcoma must, therefore, be considered in the differential diagnosis when a mass is detected in the lower extremity, especially without trauma [12].

#### 1.3.1. CT and Radiography

X-rays are usually normal, although a mass in the soft tissue context may be detected. Bone erosions may be visualized if there is a local invasion or, rarely, intralesional calcifications. A CT scan, preferably with contrast, will demonstrate a well-defined, homogeneous mass, usually in the vicinity of tendons or aponeurosis, with contrast enhancement [24,25].

#### 1.3.2. Magnetic Resonance Imaging

Magnetic resonance imaging demonstrates a localized mass in the vicinity of tendons or aponeurosis, most often with homogeneous signal intensity on T1-weighted imaging, lobulated morphology, and well-defined or focally infiltrating margins, which can, therefore, be easily mistaken for a lesion of a benign nature [24]. The possible absence of perilesional edema, and bone erosion may further favor the diagnostic orientation towards a non-aggressive lesion and compromise patient outcome [10].

Homogeneous contrast enhancement after gadolinium chelates intravenous injection, as well as macroscopic intralesional necrosis, are reported in the literature [10].

CCS, probably due to the presence of intracellular melanin, usually presents a slightly high signal intensity compared to muscle at T1-weighted MRI sequences; the signal intensity on T2-weighted imaging is high or intermediate in most cases, with possible heterogeneity [10]. However, lower signal intensity on T2-weighted imaging can sometimes be observed in melanin deposits. The fatty signal is never seen. However, De Beuckeleer et al. reported one patient with a myxoid component [10].

**Figure 2:**
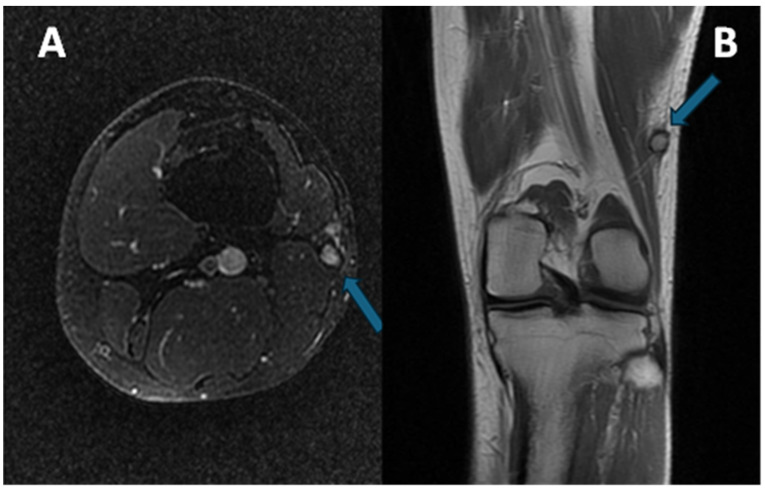
(**A**) Axial at-saturated T2-weighted imaging showing a small CCS of the left thigh (arrow): note the smooth margins, minimal perilesional edema, and deep location close to the vastus lateralis tendon. (**B**) Coronal T1-weighted imaging notes the slight hyperintensity of the T1w signal (arrow) compared to the normal muscle. A pseudocapsule, with low signal intensity, is detectable in both sequences.

Exemplificative MRI of a patient affected by CCS of the left distal thigh is provided in Figure 2.

## 2. Materials and Methods

### 2.1. Study Design

In this retrospective study, we included 14 patients (8 males and 6 females) with a histopathological diagnosis of CCS who were treated at our sarcoma reference center between 2005 and 2021 and of whom we had at least one pre-treatment MRI or CT scan of diagnostic quality.

In all the patients, the diagnosis was histologically proven after an Ultrasound-guided biopsy. In patients who underwent subsequent surgery, the diagnosis was confirmed with the analysis of the whole tumor after excision or amputation.

This retrospective observational original research was approved by the local ethics committee of our Institution; approval code CE-AVEC 47/2025/Oss/IOR, acronym RX.RARE.SARC, discussed on 23 January 2025. The research was carried out in compliance with the rules established by our local ethics committee and according to the Declaration of Helsinki and its later amendments.

### 2.2. Clinical Data Collection

A senior orthopedic oncological surgeon with 15 years of expertise examined clinical reports from our institution. Age, gender, pain upon diagnosis, tumor site, depth to superficial fascia, and early metastatic stage were among the information gathered.

When available, the outcome included the patient’s most recent state, which was classified as either dead of disease (DOD), alive with illness (AWD), dead not connected to disease (DUD), or no evidence of disease (NED).

### 2.3. Imaging Analyses

All available radiological studies (images and reports) for local and remote staging were reviewed in agreement by two radiologists (P.S. and P.D.), with 13 and 3 years of experience in musculoskeletal oncological imaging, respectively, on an image archiving and communication system (PACS-Carestream Vue PACS v. 11.4.1.1102, Philips Healthcare, Amstelplein 2, Amsterdam, The Netherlands). In each modality, the longest diameter (LD), taking into account the three planes, was measured. The surrounding structures were evaluated for involvement, either in terms of bone degradation or encasement of nerves or vessels.

#### MRI Studies and Analysis

T1-weighted imaging, T2-weighted imaging, and a fluid-sensitive sequence (T2-weighted imaging fat-saturated or short tau inversion recovery-STIR or DP fat-saturated) were always included in the protocol used for the MRI imaging experiments, which were conducted using a Signa HD, 1.5 Tesla (General Electric Healthcare, Chicago, IL, USA).

The LD was recorded on contrast-enhanced MRI sequences or CECT (in mm), in the longest axis (considering the three spatial planes).

The lesion growth pattern (categorized as regular margins/pushing type, focally infiltrating or diffusely infiltrating), signal intensity on T1-weighted imaging and T2-weighted imaging (defined as hyperintense, isointense, or hypointense compared to normal muscle), presence/absence of macroscopic intra-tumoral necrotic signal (defined as low signal intensity on T1-weighted imaging, high signal intensity on T2-weighted imaging and no contrast enhancement), and signal intensity homogeneity (on T2-weighted and on T1w-after contrast injection) were examined.

The surrounding tissues were evaluated for subcutaneous tissue invasion (absent or present), bone erosion (absent, present), tail sign (defined as thick contrast enhancement of fascia spreading from the tumor and categorized as absent, present) [26], peritumoral edema (defined as high signal intensity on T2-weighted imaging without mass effect in the tissues surrounding the tumor and categorized as absent, focal, or diffuse), and peritumoral enhancement (defined as high signal intensity on contrast-enhanced T1-weighted imaging without mass effect, in the tissues surrounding the tumor and categorized as absent or present) [27,28].

### 2.4. Statistical Analysis

A Fisher’s exact test was used to assess associations between tumor size, individual imaging features, and the risk of metastasis at baseline, at 5 years after diagnosis, and the occurrence of metastasis in general during follow-up (also beyond 5 years). For each imaging feature analyzed, the univariable odds ratio (OR) with a 95% confidence interval (CI) was also calculated to assess the risk of metastasis at diagnosis, 5 years later, and during follow-up. For the calculation of the odds ratio from contingency tables, the following approximation was applied: if one of the terms in the denominator equaled 0, it was replaced with the value 0.5 to avoid undefined results. Pearson correlation analysis and logistic regression were calculated. Statistical analyses were performed with RStudio software (RStudio 2024.12.0 Build 467). Data were considered statistically significant when *p* < 0.05. All tests performed were two-tailed.

## 3. Results

### 3.1. Clinical Data

A total of 14 patients (8 males, 6 females; mean age 39.4 years, range 14.6–66.8 years) with a histopathological diagnosis of CCS and available pre-treatment MRI or CT investigations, treated between 2005 and 2021 at our sarcoma reference center, were included.

The median follow-up was 6.7 years (range 0.13–17.06 years).

In total, five patients were treated with amputations (5/14—35.7%), and nine with surgical excision (64.3%).

Patients’ main clinical data are summarized in Table 1.

### 3.2. Metastatic Pattern

In total, 5 out of 14 patients had metastases at diagnosis (35.7%), and in a further 3 patients, metastases appeared within the first 5 years after diagnosis (1, 1.4, and 1.7 years, respectively), thus with 57.14% of patients presenting distant metastases within 5 years after diagnosis.

In 2 further patients, metastases appeared more than 5 years after diagnosis (after 6.7 and 14.6 years, respectively) for a total of 10 metastatic patients during follow-up (10/14, 71.4%).

The sites affected by metastases were the lymph nodes (7/10—70%), lungs (50%), distant soft tissue (30%), and bone (30%)—several patients were affected by metastases in multiple sites.

Patients were evaluated at baseline as well as in follow-up controls with whole-body CECT and/or PET-CT. Data on metastatic status were obtained from this imaging study’s results.

### 3.3. General Imaging Findings—Local and Distant Baseline Assessment

#### 3.3.1. Dimensional Assessment (Longest Diameter—LD)

The mean LD found at diagnosis is 50.5 mm, with a range from 16 to 110 mm.

The Pearson correlation coefficient between LD and metastasis at diagnosis is 72%, with a highly significant *p*-value of 0.004. The two variables are strongly correlated.

Logistic regression returns an estimate of the intercept coefficient of −5.26, with a *p*-value of 0.05. The coefficient of LD is 0.08, with a *p*-value of 0.05. For each millimeter increase in diameter, the probability of metastasis increases by a factor of 1.088 (approximately 8.8%). In summary, the logistic regression model confirms a positive relationship between diameter and probability of metastasis. The weakly significant *p*-value values, likely due to the small sample size, suggest further investigations to confirm the obtained results.

**Figure 3:**
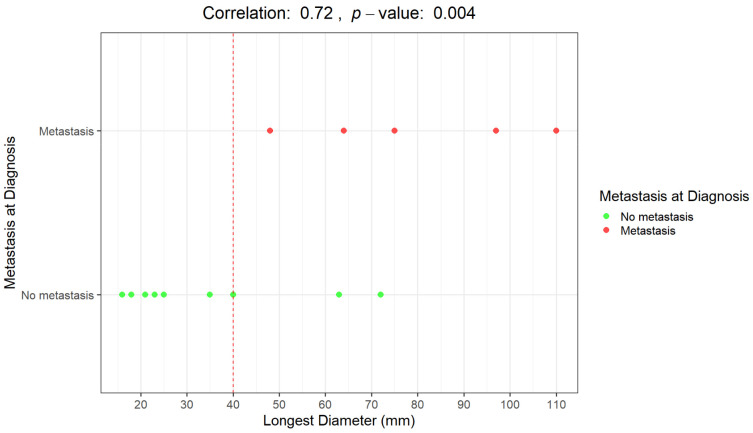
Graphical representation of the correlation between the longest diameter (mm) and the presence/absence of distant metastases at diagnosis. Distributions of patients with metastases at diagnosis (red dots) and without metastases at diagnosis (green dots).

**Figure 4:**
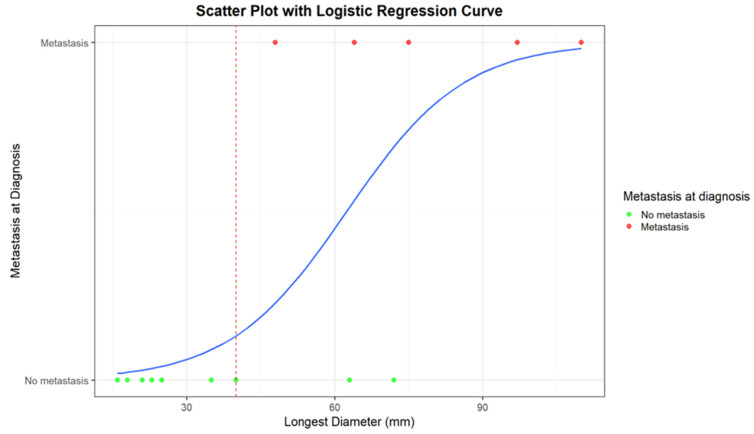
Scatter plot with logistic regression curve for the correlation between the longest diameter (mm) and the presence/absence of distant metastases at diagnosis. Patients with metastases at diagnosis are represented as red dots, while patients without metastases are represented as green dots. The blue line corresponds to the logistic regression curve, calculated using a logistic model, demonstrating the relationship between tumor diameter and the likelihood of metastases. The red dashed line indicates the cut-off diameter beyond which strong correlations between the presence of metastases and tumor diameter become evident.

In Figure 3 and Figure 4, correlations between LD distribution and metastatic disease are graphically presented.

An LD of 40 mm represents a cut-off value among the presence/absence of distant metastases at diagnosis; all the patients with metastases at diagnosis presented with a baseline LD > 4 cm. Moreover, all the patients who remained metastasis-free for 5 years after diagnosis had a maximum diameter of less than 40 mm (mean maximum diameter: 23 mm; range 16–35 mm).

The maximum diameter at diagnosis greater than 40 mm was significantly associated with the presence of metastases within the first 5 years after diagnosis (*p* = 0.0003). A maximum diameter greater than 4 cm represents a risk of metastatic disease within 5 years with an OR of 195.00 (95%CI: 3.36–11,285.55), *p* = 0.01 (Table 2 and Table 3).

Furthermore, a maximum diameter at diagnosis of more than 40 mm is also significantly associated with the occurrence of metastases in general (even beyond 5 years from diagnosis) (*p* = 0.02). It represents a significant increase in the risk of metastatic disease in general, with an OR of 30.60 (95%CI: 1.19–784.70), *p* = 0.04 (Table 4 and Table 5).

#### 3.3.2. Macroscopic Necrosis (Assessed with MRI or CECT)

The presence of macroscopic necrosis at diagnosis, assessed with contrast-enhanced MRI or CECT, was not significantly associated with the presence/absence of metastases, considering all the follow-up controls (*p* = 0.1026). However, it represents a significant increase in the risk of “early” metastatic disease within the first 5 years of follow-up with an OR of 15.0000 (95%CI: 1.0306–218.3109), *p* = 0.0475 (Table 6).

The presence of macroscopic necrosis at baseline imaging is slightly associated, even if without statistical significance, with the occurrence of metastases considering all the follow-up controls (*p* = 0.07), and it represents a not statistically significant increase in the risk of metastatic disease with an OR of 19.29 (95%CI: 0.80–466.26), *p* = 0.07.

**Figure 5:**
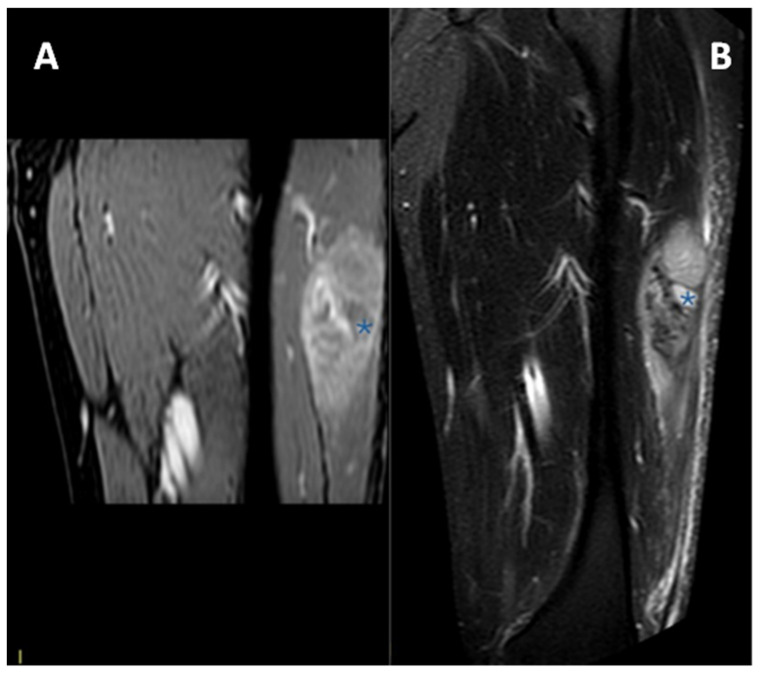
MRI in a case of deep-seated CCS of the left thigh. (**A**) T1 fat-saturated post-contrast coronal plane. (**B**) T2 fat-saturated coronal plane. A clear macroscopic area of necrosis within the lesion is appreciable (asterisks).

**Figure 6:**
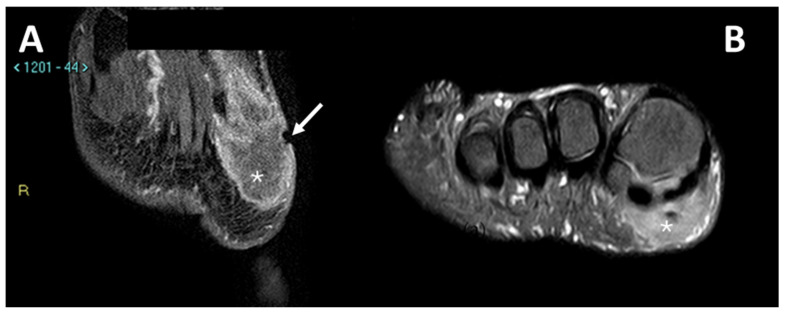
MRI in a case of CCS of the plantar region of the right foot. (**A**) Coronal contrast-enhanced fat-saturated T1-weighted imaging and (**B**) axial fat-saturated T2-weighted imaging; note the central area of macroscopic necrosis (asterisks) characterized by T2 signal hyperintensity and absence of enhancement. A slight focal infiltration in the subcutaneous tissues can be noted as well (arrow).

In Figure 5 and Figure 6, MRIs of two cases of CCS are displayed, both with macroscopic areas of necrosis detectable.

### 3.4. MRI Features

#### 3.4.1. General MRI Features

**Figure 7:**
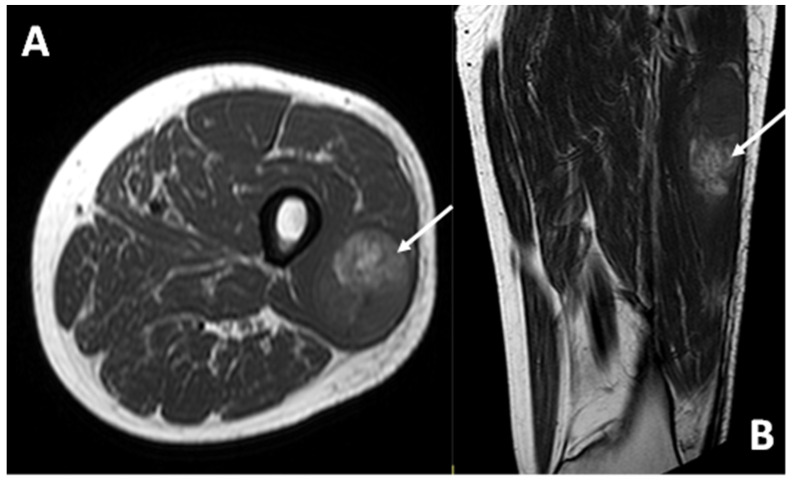
T1-weighted sequences in axial (**A**) and coronal (**B**) planes of a deep-seated CCS of the thigh: note the high signal intensity of the lesion (arrows) compared to the normal adjacent muscle, the deep location, and the smooth margins.

In all cases examined with MRI (13/13), the lesion demonstrated a hyperintense signal intensity on T1w sequences compared to healthy muscle (Figure 7).

In all cases (13/13), the CCS was hyperintense in the T2 sequences with varying degrees of signal heterogeneity.

The growth pattern was non-infiltrating (pushing type) in six cases (46%), focally infiltrating in five cases (38%), and diffusely infiltrating in one case (8%). Intralesional macroscopic necrosis was present, to varying degrees, in seven patients (7/14—50%). A sort of pseudocapsule with very low signal intensity is appreciable at the borders (partial or complete) of the lesions in almost half of patients with MRI available (6/13 = 46%).

CCS presented deep subfascial localization in 10 cases (71%) and superficial in 4 cases (29%). Three of the patients with deep lesions (3/10, 30%) had associated bone erosion.

Diffuse peritumoral edema was present in six cases (42%), while in five cases, it was only focal (35%), and in two cases, it was absent (14%). Peritumoral contrastographic enhancement was found in three cases (21%).

A tail-sign was found in only one patient (7%).

In cases where contrast media was administered (11 out of 13 patients), impregnation covered 75–100% of the lesion in nine cases (64%), while in the remaining two cases (14%), less than 50% of the lesion showed contrast enhancement due to the presence of larger areas of necrosis.

#### 3.4.2. Peritumoral Edema

The following results for peritumoral edema are not significant from a statistical point of view, given the cut-off of 5% for the *p*-value. However, the results were near the significant value and, considering the small number of subjects, we decided to report them.

The presence of diffuse peritumoral edema, assessed with MRI (on fluid-sensitive sequences), was not significantly associated with the presence of metastases within 5 years (*p* = 0.1026). The presence of peritumoral edema is associated with an increased risk of metastatic disease within 5 years in our sample, even if without a statistically significant value, with an OR of 12.5000 (95%CI: 0.8387–186.3082), *p* = 0.0669.

The presence of diffuse peritumoral edema was not significantly associated with the occurrence of metastases during follow-up (*p* = 0.07) nor did it represent a significant increase in the risk of metastatic disease in general in our sample with an OR of 16.71 (95%CI: 0.68–409.12), *p* = 0.08.

#### 3.4.3. Other MRI Features

All the other MRI features assessed were not associated with metastatic disease at diagnosis and presented lower OR values and higher *p*-values as metastatic risk factors, compared with the previously mentioned features (dimensions, necrosis, and peritumoral edema).

## 4. Discussion

CCS is an extremely rare melanocytic sarcoma of the soft parts [29]. At diagnosis, it is usually a relatively small (<5 cm), deep lesion, often juxtaposed to tendons, fascia, or aponeurosis, often with non-infiltrating margins (even if bone erosion may occur), most frequently affecting the lower limbs and showing slow, indolent growth. In this original series, we performed exhaustive depictions of the radiological features of CSS and their association with metastatic disease. We demonstrated that size (LD > 4 cm), necrotic signal on MRI, (and slightly peritumoral edema even if without statistically significant values) were correlated with the patient’s risk of metastatic relapse.

The current research has several limitations; first of all, the small number of patients included (due to the rarity of this diagnosis). Moreover, the retrospective nature of the research represents an intrinsic limit. Nonetheless, the imaging studies available were not homogeneous (13 MRIs and 1 CECT). We did not analyze the value of PET results (e.g., SUV values related to prognosis) because of the small number of subjects with this imaging tool available at the baseline. The study was focused on imaging features only, and an analysis of clinical/surgical/histological findings (e.g., tumor margins) was not included in the current research. A multivariate analysis considering the various independent variables examined would have provided valuable insights. Unfortunately, the limited number of observations in the dataset did not allow for statistically significant results to be obtained.

Our analysis confirms that the size of the lesion at the baseline MRI is a key element for patients’ prognosis prediction, reflecting the fact that an early diagnosis and fast referral to sarcoma centers is crucial. This was confirmed by previous research for all STS subtypes, even for the rarest and atypical ones [30,31].

All the cases presented received a histopathological diagnosis of clear cell sarcoma and molecular confirmation of the presence of the typical transcription gene (EWSR1/XX).

We presented the radiological characteristics of CCS patients treated at our sarcoma referral center over a 16-year period (2005–2021). Our series, although limited in number, represents a significant radiological experience in the context of ultra-rare sarcoma, especially with regard to MRI imaging characteristics.

All patients had at least one CT or MRI at the baseline, as per inclusion criteria.

In our series, the MRI features consistently found were (i) high signal intensity at T1-weighted imaging compared to healthy muscle, of variable degree and probably due to the presence of intracellular melanin; (ii) high signal intensity at T2-weighted imaging (though not fluid-like) [10]. All lesions exhibited a variable degree of signal heterogeneity on T2 images.

Signal hyperintensity in T1 is quite rare among soft tissue lesions and, when present, drastically narrows the field of differential diagnosis to lesions of a lipomatous nature (lipoma, well-differentiated liposarcoma, lipoblastoma), which can be differentiated from CCS by fat-suppressed sequences; alveolar soft part sarcoma or malignant solitary fibrous tumor, which, however, will have additional peculiar features that are absent in CCS (flow voids, a high T2 signal); melanocytic schwannoma, which is located near nerve roots or the course of peripheral nerves, and subacute phase hematoma; may have similar signal characteristics but can be differentiated from CCS by the absence of enhancement [30]. In this regard, it is important to underline that MRI evaluation of a soft tissue tumor does not have the goal of offering a histological diagnosis, but of suspecting the potential of malignancy (aggressive biological behaviors) and suggesting further examinations and rapid referral to sarcoma centers [32].

**Figure 8:**
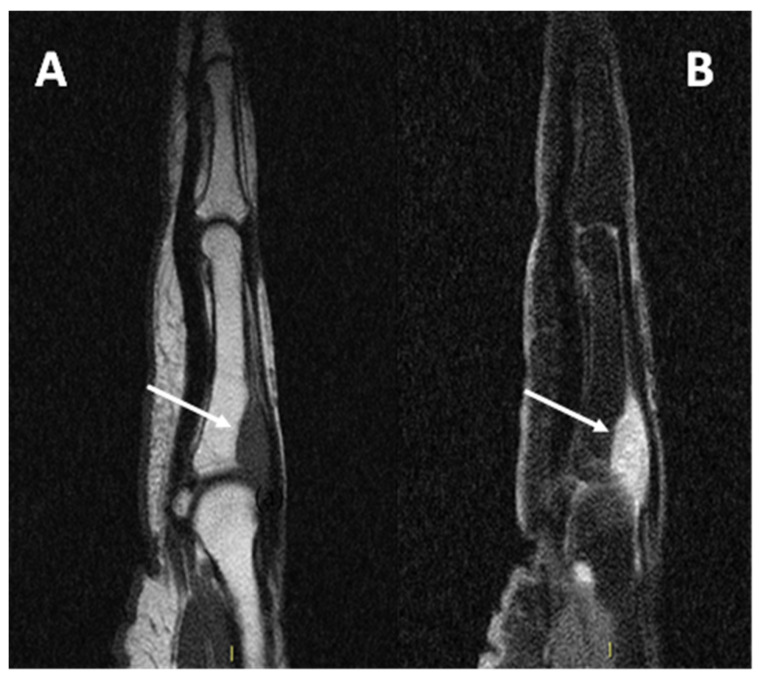
(**A**) sagittal T1w sequence (**B**) sagittal T1w fat-saturated post-contrast media injection of a CCS of the second metacarpophalangeal joint: note the bone erosion on the dorsal aspect of the proximal phalanx (arrows).

Due to its close relationship with tendons and aponeurosis (Figure 8), CCS may also be difficult to differentiate from a tendon sheath giant cell tumor (TCG), which typically affects the hands and may present an intermediate signal at T1 due to the presence of hemosiderin [15].

The absence of an anatomopathological grading for this particularly rare tumor renders the identification of radiological features correlated to prognosis even more important.

Some of the lesions examined show a non-infiltrating growth pattern (pushing type, sometimes with pseudocapsule), in the minority focally infiltrating and only in one case diffusely infiltrating. Diffuse perilesional edema is also an inconstant feature, which in our series does not correlate with the risk of metastasis. Bone erosion and invasion of superficial planes can sometimes be detected. The combination of these features and the inconstant presence of imaging signs of clear malignancy lead to serious delays in the recognition of the pathology and its aggressiveness.

In our case series, several patients underwent inadequate excision of the lesion, and only after histological diagnosis were they referred to our center for appropriate treatment. Recent original research focused on CCS revealed that the surgical margins significantly influenced the prognosis. Patients with radical margins (R0) presented significantly (*p* = 0.017) longer survival rates compared to R + status in localized CCS (N0M0, 5-yr OS 0% vs 64%) [33].

**Figure 9:**
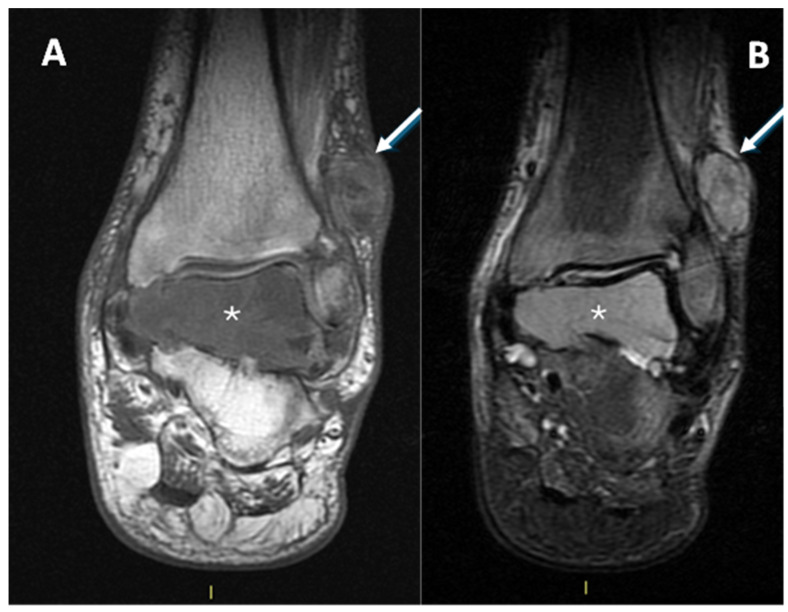
(**A**): T1w coronal sequence, (**B**): STIR coronal sequence. CCS arising from the peroneal tendons (arrows) in the context of neuropathic diabetic foot and bone metastasis at the talus (asterisks). A partial pseudo-capsule, with low signal intensity, is appreciable on the periphery of the primary tumor of the soft tissue.

One patient presented with metastases at diagnosis because the lesion arose in the context of a diabetic foot, delaying the correct histological diagnosis by several years (Figure 9).

Considering the malignant nature of the lesion and its non-aggressive appearance on imaging, the identification of distinctive features that make CCS recognizable may increase the patient’s chances of receiving appropriate treatment.

In our case series, a diameter of more than 4 cm correlates significantly with the presence of metastases within 5 years of diagnosis. Therefore, recognition of the lesion at an early stage plays a key role in patient outcomes.

Other imaging features associated with the worst outcome (distant metastases) in our series are signs of macroscopic necrosis. This feature is already recognized as a key element for prognosis prediction in other miscellaneous STS research [27,28,34].

Knowledge of the characteristics of CCS, especially its hyperintensity at T1, and the identification of such lesions as malignant are crucial to referring patients to a biopsy characterization and a specialized center for appropriate treatment [32].

## 5. Conclusions

The imaging features found in our study are in line with the limited available literature on the subject [10]. In our case series, the significance of the correlation of certain features with prognosis (size > 4 cm and the presence of intralesional macroscopic necrosis) underlines the importance of early recognition of these lesions as malignant and the central role of imaging in referring the patient to a specialized center as early as possible.

Future multi-institutional research on this topic is desirable for an even better definition of its characteristics and for the identification of further potential diagnostic parameters correlated with prognosis.

## Figures and Tables

**Table 1:** Patients’ main characteristics, imaging studies available, tumor sizes, and metastatic presence at diagnosis, within 5 years, and during follow-up.

Patient n°	Age, Sex	Symptoms	Lesion Location (Deep/Superficial)	Longest Diameter (mm)	Metastasis at Diagnosis	Metastasis Within 5 Years	Metastasis During F-U
1	31, 8 M	Lump	Left thigh, deep	23	No	No	**Yes**
2	31 M	Lump	Left foot, superficial	48	**Yes**	**Yes**	**Yes**
3	39, 3 F	Lump	Right foot, deep	21	No	No	**Yes**
4	60, 5 F	Lump	Left hand, deep	25	No	No	No
5	37, 6 M	Lump	Right knee, deep	35	No	No	No
6	47, 2 M	Painful lump	Left thigh, deep	110	**Yes**	**Yes**	**Yes**
7	66, 8 M	Painful lump	Left foot, deep	64	**Yes**	**Yes**	**Yes**
8	34, 3 M	Painful lump	Left foot, deep	97	**Yes**	**Yes**	**Yes**
9	58, 1 F	Lump	Left foot, deep	40	No	**Yes**	**Yes**
10	33, 9 M	Lump	Right foot, deep	72	No	**Yes**	**Yes**
11	17, 7 F	Lump	Right foot, superficial	18	No	No	No
12	14, 6 F	Lump	Right foot, superficial	16	No	No	No
13	42, 1 F	Lump	Left shoulder, deep	75	**Yes**	Yes	**Yes**
14	36, 4 M	Lump	Perineum, superficial	63	No	**Yes**	No

**Table 2:** Imaging characteristic (diameter > 4 cm) and correlation with the occurrence of metastases within 5 years.

Imaging Feature	M0 < 5 Years	M1 < 5 Years	*p*-Value
<4 cm	6	0	
≥4 cm	0	8	**0.0003**

**Table 3:** Imaging characteristic (diameter > 4 cm) and risk of metastasis within 5 years from diagnosis (Odds Ratio—OR, univariate analysis).

Imaging Feature	M0 < 5 Years	M1 < 5 Years	Odds Ratio (95% CI)	*p*-Value
>4 cm	0/6	8/8	195.00 (3.37–11,285.55)	**0.01**

**Table 4:** Imaging characteristic (diameter > 4 cm) and correlation with the occurrence of metastases during follow-up.

Imaging Feature	M0	M1	*p*-Value
<4 cm	4	2	
>4 cm	0	8	0.02

**Table 5:** Imaging characteristic (diameter > 4 cm) and risk of metastasis (Odds Ratio—OR, univariate analysis).

Imaging Feature	M0	M1	Odds Ratio (95% CI)	*p*-Value
>4 cm	0/4	8/10	30.600 (1.19–784.70)	**0.04**

**Table 6:** Imaging characteristic (presence of necrosis) and risk of metastasis within the first 5 years of follow-up (Odds Ratio—OR, univariate analysis).

Imaging Feature	M0 < 5 Years	M1 < 5 Years	Odds Ratio (95% CI)	*p*-Value
Necrosis	1/5	6/8	15.00 (1.03–218.31)	**0.05**

## Data Availability

The data presented in this study are available on request from the corresponding author.
